# Supplementary material for: Controlled and customizable baculovirus NOS3 gene delivery using PVA-based hydrogel systems
Source: PLoS One. 2023 Sep 21;18(9):e0290902. doi: 10.1371/journal.pone.0290902 (PMC10513238; doi:10.1371/journal.pone.0290902)
Supplement: Attachment — Submitted filename: PONE-D-23-10026 reviewer responses.docx [file pone.0290902.s002.docx]

**PONE-D-23-10026 Editor & Reviewer Responses**

Controlled and customizable baculovirus NOS3 gene delivery using PVA-based hydrogel systems

**Dear Editor:**

Thank you for your advice and recommendations. We appreciate the opportunity to revise manuscript. We have followed and completed all your recommendations. Briefly:

1. We have reformatted the manuscript and figure files to meet PLOS One’s requirements.
2. The funding information has been checked and should be as follows:
   a. This work was supported by a grant from the Canadian Institute of Health Research (CIHR, grant 252743) to Dominique Shum-Tim and Satya Prakash. S.S. is fully funded by the Canadian Graduate Scholarship-Doctoral from the Natural Sciences and Engineering Research Council (NSERC, 569661-2022).). P.I. is funded by the Islamic Development Bank Scholarship (2020-245622). A.A. is fully funded by a scholarship from the Ministry of Higher Education of the Arab Republic of Egypt. R.T. and K.A. are funded by the Canadian Graduate Scholarship-Master’s from NSERC. The funders had no role in study design, data collection and analysis, decision to publish, or preparation of the manuscript.
3. The statement has been added after the funding information provided in the manuscript.
4. The competing interests form has been completed, <http://journals.plos.org/plosone/s/submit-now>
5. Data availability statement: the minimal data underlying the results has been provided in the attached Excel sheet with each figure labelled.
6. Ethics approval was not required for the study and this statement has been added to the Methods section of the manuscript.

**Reviewer #1**

**Comment:** The study provides important insights into the potential of NOS3 eluting PVA-based hydrogels for medical applications and device coatings. The addition of gelatin to the hydrogels is shown to be effective in protecting baculovirus viability and enabling sustained release. However, the study has limitations in terms of investigating the actual effects of NOS3 delivery on cell proliferation, blood vessel vasodilation, and bacterial growth inhibition, as well as providing detailed characterization of the properties of the hydrogels. Nevertheless, the study has value in providing a proof of concept for a potential delivery system and highlights areas for further research and development.

The study only investigates the release of baculoviruses and their potential for NOS3 gene delivery. It does not investigate the actual effect of NOS3 delivery on cell proliferation, blood vessel vasodilation, or bacterial growth inhibition.

The study only investigates the effect of hydrogel composition on properties such as elasticity, blood coagulation, and antimicrobial effects, but does not provide a detailed characterization of these properties.

The study does not investigate the long-term stability of the hydrogels or the effect of storage conditions on their properties and performance.

**Response:** Thank you for your approval of the manuscript. We also appreciate your important suggestions. As per your suggestions and recommendations, we have revised this manuscript. You will find we have addressed all your concerns. Briefly:

The effect of NOS3 delivery on both HUVEC and SMC proliferation is presented in Figure 4e-f. We have also added a Griess assay to measure nitrate concentration (Figure 4d). An antibacterial assay using NOS3 and nitrate has been added. Moreover, previous papers have already established NOS3 and nitric oxide as a cardiovascular support and antimicrobial properties and have been cited in lines 470-478.

Further details have also been added for the hydrogel swelling, antimicrobial effects, and blood coagulation on pages 13-14.

The long-term stability was added to lines 456-460 and 334-335.

Storage conditions of the baculovirus-eluting hydrogels have been added to lines 456-460.

**Reviewer #2:**

This manuscript evaluates the use of hydrogel formulations for sustained delivery of baculovirus in a proof-of-concept study. The manuscript is well written, properly illustrated and appropriately referenced. The result stand on their own. Baculovirus as a vehicle for gene delivery and gene editing is increasingly gaining interest and traction due to the numerous advantages of this viral vector above the state-of-the-art. This study represents and interesting and insightful contribution that will be valuable for the field.

**Response:** Thank you for your acceptance and recommendation of the article for publication.
